# Unleashing the Potential of CRISPR/Cas9 Genome Editing for Yield-Related Traits in Rice

**DOI:** 10.3390/plants13212972

**Published:** 2024-10-24

**Authors:** Archana Thiruppathi, Shubham Rajaram Salunkhe, Shobica Priya Ramasamy, Rakshana Palaniswamy, Veera Ranjani Rajagopalan, Sakthi Ambothi Rathnasamy, Senthil Alagarswamy, Manonmani Swaminathan, Sudha Manickam, Raveendran Muthurajan

**Affiliations:** 1 Department of Plant Biotechnology, Centre for Plant Molecular Biology and Biotechnology, Tamil Nadu Agricultural University, Coimbatore 641003, India; tarchanaagri@gmail.com (A.T.); shubhamsalunkhe254@gmail.com (S.R.S.); rakshanapalanisamy@gmail.com (R.P.); rajaranji@gmail.com (V.R.R.); arsakthy@gmail.com (S.A.R.); 2 Department of Plant Breeding and Genetics, Centre for Plant Breeding and Genetics, Tamil Nadu Agricultural University, Coimbatore 641003, India; ramshoby20@gmail.com; 3 Department of Crop Physiology, Tamil Nadu Agricultural University, Coimbatore 641003, India; senthil.a@tnau.ac.in; 4 Department of Rice, Centre for Plant Breeding and Genetics, Tamil Nadu Agricultural University, Coimbatore 641003, India; manonmanitnau@gmail.com

**Keywords:** genome editing, CRISPR/Cas9, rice, grain yield, yield genes

## Abstract

Strategies to enhance rice productivity in response to global demand have been the paramount focus of breeders worldwide. Multiple factors, including agronomical traits such as plant architecture and grain formation and physiological traits such as photosynthetic efficiency and NUE (nitrogen use efficiency), as well as factors such as phytohormone perception and homeostasis and transcriptional regulation, indirectly influence rice grain yield. Advances in genetic analysis methodologies and functional genomics, numerous genes, QTLs (Quantitative Trait Loci), and SNPs (Single-Nucleotide Polymorphisms), linked to yield traits, have been identified and analyzed in rice. Genome editing allows for the targeted modification of identified genes to create novel mutations in rice, avoiding the unintended mutations often caused by random mutagenesis. Genome editing technologies, notably the CRISPR/Cas9 system, present a promising tool to generate precise and rapid modifications in the plant genome. Advancements in CRISPR have further enabled researchers to modify a larger number of genes with higher efficiency. This paper reviews recent research on genome editing of yield-related genes in rice, discusses available gene editing tools, and highlights their potential to expedite rice breeding programs.

## 1. Introduction

The rising global population poses a significant challenge to ensuring food security, demanding a collective effort to enhance productivity. Rice, being one of the major food crops, plays a pivotal role in providing nourishment for over fifty percent of the world’s population. To meet the increasing nutritional requirements, qualitative traits of rice like culm habits and quantitative traits such as grain yield, grain size, etc., must be improved [1]. Rice is also adopted as a model system in plant science due to its compact genome size, well-established molecular marker linkage maps, and the availability of efficient transformation technologies [2].

Various approaches have been utilized to enhance rice quality and quantity. Conventional breeding methods, such as mutational breeding and hybridization methods, have been used to improve rice varieties; however, they are considered tedious, time-consuming and inclined to human bias [3]. These approaches may introduce undesirable genes alongside targeted genes, and hybridization is restricted to plants within the same species. While other techniques like genetic engineering and molecular approaches have also shown promise in enhancing crop varieties, they come with certain limitations as well, such as ethical concerns, potential environmental risks, genetic instability, etc. In recent years, genome editing technologies have addressed the constraints associated with traditional breeding methods and can quickly introduce desirable traits into any plant species including rice, wheat, barley, cowpea, chickpea, cotton, tomato, etc., in a short time. Consequently, they hold significant potential to accelerate breeding programs [4].

Genome editing has ushered in a new era of genome engineering, facilitating the efficient and accurate modification of plant genomes in a remarkably short time frame [5]. The technique relies on site-specific nucleases (SSNs), encompassing mega-nucleases, zinc finger nucleases (ZFNs), transcription activator-like effector nucleases (TALENs), and clustered regularly interspaced palindromic repeats (CRISPRs)/CRISPR-associated-Cas9 systems. These nucleases are utilized to induce double-strand breaks (DSBs) at targeted sites of DNA. The desired mutations can then be generated through the endogenous, error-prone non-homologous end joining (NHEJ) or homology-directed repair (HDR) pathway. Therefore, precise modifications to a target gene, including specific substitutions, insertions and deletions of a desired sequence, can be carried out with greater accuracy [6,7].

The CRISPR/Cas9 system is considered an effective genome editing tool due to its potential to generate novel modifications to a target gene with relative ease and high efficiency [8]. Another significant benefit of the CRISPR/Cas9 system is its ability to target multiple genes or specific sites within a gene, facilitating the creation of small or large deletions in the genome [9]. CRISPR/Cas9 has proven to be highly effective in enhancing agricultural traits, particularly agronomic traits, which ultimately result in increased crop yield. This review delves into recent genome editing research focusing on genes associated with yield traits and their alterations to enhance rice crop improvement. Additionally, it mentions the challenges posed by genome editing technologies and explores potential solutions to surpass them.

## 2. Yield and Its Component Traits

**Figure 1:**
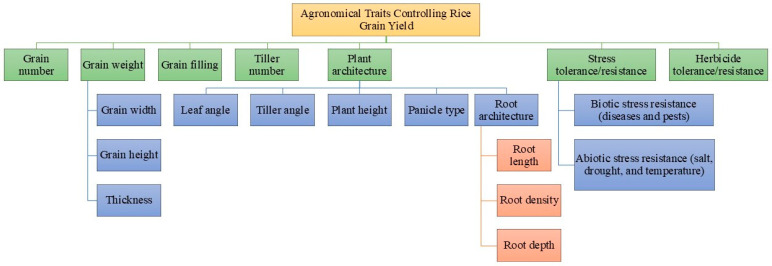
Key agronomic traits contributing to rice grain yield.

Grain yield, the culmination of various agronomic factors and genetic traits, stands as a foremost aim of rice agriculture and is influenced by numerous genes operating within complex signaling pathways. It can be determined by three primary traits: number of grains per panicle, number of panicles per plant (tillering ability of a plant), and grain weight [2]. Plant height can indirectly influence grain yield by affecting the ability of plants to absorb sunlight and compete for resources with neighboring plants [10]. Aside from these components, factors such as grain filling, panicle architecture, and leaf orientation play crucial roles in determining the rice grain yield (Figure 1). The extent and rate of grain filling are predictive of the final grain weight and are essential for both the grain quality and yield [11]. Nitrogen use efficiency (NUE) and photosynthetic efficiency are two physiological traits that greatly impact grain yield [12]. Phytohormone signaling, stress tolerance, diverse pathways for plant growth and developmental and transcriptional regulation are additional factors that could potentially influence rice grain yield. Therefore, agronomic traits, such as plant architecture (leaf angle, tiller angle, tiller number, plant height, panicle type, etc.) and grain formation (grain number, grain weight and grain filling), and the above-mentioned physiological traits are key factors that need to be considered to enhance the productivity of rice [13].

## 3. Agronomical Traits

### 3.1. Genes Controlling Rice Grain Number

The number of grains per panicle is an important trait for rice yield potential. It is primarily influenced by the type of panicle and the differentiation of branches, which are closely linked to various phytohormone pathways and vascular differentiation [14,15]. Studies have revealed that numerous genes from these pathways hold considerable promise for enhancing grain number and ultimately improving rice grain yield [16]. *Gn1a* codes for the enzyme cytokinin oxidase/dehydrogenase (OsCKX2), which is responsible for the degradation of cytokinin. When the expression level of OsCKX2 decreases, cytokinin levels increase in the inflorescence meristems, leading to a better reproductive organ and eventually resulting in a higher yield [17]. The CRISPR/Cas9 construct, aimed at targeting *Gn1a*, resulted in frame-shift mutants in Zhonghua 11 (rice cultivar) and showed increased plant height, panicle size and flower number per panicle [18].

“Seed size/seed number trade-off” is a well-known phenomenon observed not only in rice but also in many plant species. There exists a negative correlation between the size of seeds and their number, which is attributed to metabolic constraints [19]. When plants have limited resources available for reproduction, if they invest more resources to produce a larger number of seeds, then the seed size will tend to decrease, and vice versa [20]. The *GSN1* gene encodes the mitogen-activated protein kinase (MAPK) phosphatase enzyme (OsMPK1); it is found to be “the molecular brake” precisely governing both grain size and grain number and changes in the expression of the gene directly influence the panicle type and grain shape. The characterization of the rice mutant *grain size and number1 (gsn1)* highlights that they exhibit larger grains but are fewer in number compared to the wild type due to the presence of disordered localized cell differentiation and proliferation. Thus, to put it another way, *GSN1* promotes grain number but inhibits grain size. *GSN1* directly interacts with OsMPK6 and inactivates the enzyme via dephosphorylation. Suppression of the MAPK cascade (OsMPKK10-OsMPK4-OsMPK6) genes (via CRISPR/Cas9 and RNAi), which participate in panicle morphogenesis, resulted in denser panicles with reduced grain size, thus effectively alleviating the phenotypes observed in the *gsn1* mutant. Hence, *GSN1* also acts as a negative regulator of the MAPK cascade, which influences the panicle architecture. Ultimately, the validation of the GSN1-MAPK module confirms its role in coordinating the trade-off between grain number and grain size in rice [21]. Another gene, *OsPUB3*, appears to coordinate the trade-off between grain weight and yield. When the *OsPUB3* gene was knocked out, it reduced the grain weight but increased the number of grains per panicle and the number of panicles per plant, thus stabilizing the overall yield of a plant [22].

Spikelet number in a panicle is another important factor which influences the number of grains per panicle. Recently, it has been documented that the number of spikelets in a panicle can be controlled by modulating the expression level of Floral Organ Number (*FON4*). It was reported that fon4, a mutant allele generated via the CRISPR/Cas9 system, is responsible for forming lateral florets alongside the normal terminal florets. Thus, mutations in the *FON4* gene have the capability to induce the formation of spikelets with multiple flowers, consequently resulting in the production of multiple seeds [23].

The 5′ untranslated region (UTR) significantly contributes to the regulation of gene expression [24]. *FRIZZY PANICLE (FZP)* encodes an AP2/ERF (ethylene response factor) transcription factor and is involved in the formation of panicle branching in rice [25]. The deletion of a specific 113bp segment (−157 to −45bp in UTR) upstream of the *FZP* gene showed increased grain numbers in rice [26].

*DENSE ERECT PANICLE (DEP1),* encoding the G protein ẟ subunit, has been a crucial trait in rice crop improvement due to its advantageous characteristics such as high yield, lodging tolerance conferred by robust stems, and efficient nitrogen utilization. *DEP1* plays an important role in regulating panicle architecture, grain number, nitrogen absorption and stress tolerance through the G protein signaling pathway [27]. Mutants of *DEP1* exhibit a modified plant architecture characterized by a reduced plant height and grain size, dense erect panicle, and increased grain number and density [18,28]. Table 1 lists additional genes governing the rice grain number, analyzed by the CRISPR/Cas9 system, in addition to those already stated.

### 3.2. Genes Controlling Rice Grain Weight

Grain weight in rice is predominantly influenced by grain size, which is defined by three dimensions: grain length, width and thickness, as well as the extent of filling [2]. These characteristics exert such a profound impact on grain weight that they all exhibit potential trade-offs and collectively control both the grain quality and quantity [31]. Recent advances have revealed numerous pathways that significantly influence both grain number and size, including G-protein signaling; the ubiquitin-proteasome degradation pathway; biosynthesis and signaling of brassinosteroids, cytokinins, and auxins; MAPK signaling; photosynthetic product accumulation; epigenetic pathways; and transcriptional regulation [32,33]. Therefore, manipulating the genes that participate in the above-mentioned pathways is an effective strategy for improving grain size and number, which eventually enhances rice grain yields.

*GRAIN SIZE 3 (GS3)* was the first major QTL (Quantitative Trait Locus) identified to influence the grain size and quality in rice [34]. *GS3* encodes the γ subunit of heterotrimeric G-protein, which regulates cell proliferation and expansion of spikelet hull via the G-protein signaling pathway, thereby limiting grain elongation in rice [35]. The *GS3* protein consists of four major domains: a plant-specific organ size regulation (OSR) domain located at the N-terminus, a transmembrane domain, a cysteine-rich domain from the tumor necrosis factor receptor/nerve growth factor receptor (TNFR/NGFR) family, and a von Willebrand factor type C (VWFC) located at the C-terminus. These domains play distinct roles in the regulation of grain size [34]. It has been reported that the gs3 allele, featuring a mutation in the OSR domain of the *GS3* protein, leads to the production of longer grains. Conversely, a frameshift mutation lacking the TNFR and VWFC domains yielded the shortest grains, suggesting potential antagonistic effects of these domains in regulating grain size [34,36]. The frameshift mutants in the rice cultivar Zhonghua 11, generated via the CRISPR/Cas9 system, exhibited a substantial increase in grain size and had elongated awns on the husks compared to the wild type [18]. Thus, *GS3* functions as a negative regulator of rice grain size [37,38].

The *GW5* gene, encoding a calmodulin binding protein, exerts a greater impact on grain width and weight by influencing the brassinosteroid (BR) signaling pathway [39]. The *GW5* protein localizes within the plasma membrane and interacts with *GSK2* (glycogen synthase kinase2), effectively inhibiting its activity. This consequential interaction leads to the buildup of OsBZR1 (*Oryza sativa* BRASSINAZOLE RESISTANT1) and DLT (DWARF AND LOW TILLERING) proteins in their unphosphorylated states within the nucleus, facilitating the mediation of the BR signaling pathway. Ultimately, this accumulation activates genes involved in the pathway, thereby facilitating the growth responses, including grain width and grain weight in rice. Thus, *GW5* acts as a positive regulator of BR signaling and knocking out the gene significantly increased grain weight and width [40]. Another gene, *GW5L*, a homolog of *GW5,* negatively regulates grain width and size in rice [41].

*qTGW3* is another QTL that governs grain size and weight in rice. *qTGW3* encodes OsSK41/OsGSK5, a member of the GLYCOGEN SYNTHASE KINASE3/SHAGGY-like family. *OsSK41* effectively interacts with and activates *OsARF4 (AUXIN REPONSE FACTOR 4)*, a gene involved in rice grain development. Loss-of-function of both *OsSK41* and *OsARF4* has resulted in a larger grain size in rice. This suggests that *OsARF4* and *OsSK41* might operate in the same pathway regulating grain size, and their expression patterns are also notably similar. However, further investigation is required to determine if the function of *OsSK41* in grain development is entirely reliant on *OsARF4* [42].

In another study, CRISPR/Cas9-based editing was used to generate two superior alleles of *OsSPL4*, which increased the grain number and the grain size, resulting in higher rice yield. *OsSPL4* was also found to influence spikelet development by stimulating cell division, leading to the upregulation of various cell cycle and MADS-box genes in the mutated lines. Analysis of the co-expression network unveiled the involvement of numerous yield-related genes in the regulatory network of *OsSPL4.* Furthermore, *OsSPL4* was observed to undergo cleavage by os-miR156 in living organisms, suggesting that the OsmiR156-OsSPL4 module may play a role in controlling rice grain size. In addition to the aforementioned genes, Table 2 lists a number of other genes that govern rice grain weight that have been analyzed by CRISPR/Cas9.

### 3.3. Genes Controlling Rice Tiller Number

Tillering in rice plants constitutes a fundamental determinant of grain yield and serves as a model system for investigating branching in monocotyledonous plants [64]. Additionally, it is an essential agronomic characteristic that governs tolerance to plant density and resistance to lodging [13]. Tillering formation can be divided into two process: the formation of a new AM (axillary meristem) on the leaf axil and its subsequent growth [65]. Each tiller has the ability to generate a panicle. Nonetheless, tillers that appear later in the growing season demonstrate incomplete grain filling, leading to an increase in straw biomass alone. Hence, it is essential to carefully control the number of tillers to maximize potential yield [10].

*MONOCULM 1* (*MOC1*) and *MONOCULM 3/TILLERS ABSENT 1/STERILE AND REDUCED TILLERING 1* (*MOC3/TAB1/SRT1*) are two important genes that are essential for the development of tillers in rice. They interact with *FLORAL ORGAN NUMBER1 (FON1)*, the rice homolog of the *CLAVATA1* gene, which regulates tiller bud outgrowth in rice. *MOC3* directly engages with the promoter of *FON1*, facilitating its expression. In contrast, *MOC1* does not establish a direct interaction with *FON1*; rather, it functions as a co-activator of *MOC3* to promote *FON1* expression. Mutants of *MOC1* and *MOC3* showed significantly reduced expression of *FON1* in axillary meristems. Therefore, *MOC1* and *MOC3* genes positively regulate tillering in rice [66,67]. Genes governing rice tiller number, which have been examined using CRISPR/Cas9, are listed in Table 3.

### 3.4. Genes Controlling Biotic and Abiotic Stress Resistance in Rice

Worldwide, it is estimated that crop yield losses due to plant diseases range from 20% to 50% [77]. These losses result from various pathogens, including fungi, bacteria and viruses, and they considerably affect global agricultural productivity. The pathogens *Magnaporthe oryzae* and *Xanthomonas oryzae* pv. *oryzae* are responsible for causing rice blast and bacterial blight, respectively, both of which are highly destructive diseases that significantly reduce rice yields [78]. Additionally, abiotic stresses, including drought, salinity and extreme temperatures, are significant factors that hamper crop growth and development, further contributing to yield losses. Therefore, improving resistance to both biotic and abiotic stresses, using advanced techniques like CRISPR-Cas9, has proven to be the most efficient and cost-effective strategy for managing various plant stresses, ensuring sustainability and enhancing crop quality [79].

In a study, CRISPR/Cas9 was used for multiplex gene editing to simultaneously mutate three blast-resistant genes, such as *Bsr-d1, Pi21 and ERF922,* in an indica rice line (LK638S). The resulting single and triple mutants exhibited enhanced resistance to rice blast, with *erf922* mutants showing the strongest resistance. Moreover, *Pi21* and *ERF922* mutants demonstrated improved resistance to bacterial blight. This increased resistance was attributed to the upregulation of salicylic acid (SA) and jasmonic acid (JA) pathways. Notably, the mutations had no adverse effects on key agricultural traits, highlighting the effectiveness of multiplex gene editing for developing disease-resistant rice varieties [80].

Abiotic stress adversely impacts plant growth and crop productivity by disrupting various biochemical, morphological, and physiological processes that are essential for plant development [81]. Targeting the OsPUB7 gene (a Plant U-box gene in Oryza sativa) with CRISPR/Cas9 resulted in increased resistance to drought and salinity stress in rice [82]. The *OsPQT3* knockout mutants (*ospqt3*) demonstrate enhanced resistance to oxidative and salt stress, along with significantly improved agronomic performance, yielding more than the wild type when exposed to salt stress [83]. Additionally, several genes that contribute to biotic and abiotic stress resistance in rice, which have been studied using CRISPR-Cas9, are listed in Table 4.

### 3.5. Genes Controlling Herbicide Resistance in Rice

Another crucial trait that can significantly influence rice yield is herbicide resistance. The ability of rice plants to tolerate herbicides enables more efficient weed control, preventing weeds from competing for essential resources such as water, light and nutrients. Weeds are a major yield-limiting factor in rice production, often causing substantial yield losses if not effectively managed [97]. Moreover, herbicide-resistant rice varieties contribute to more sustainable farming practices by reducing the need for excessive herbicide applications and labor-intensive weed management techniques. Numerous studies have highlighted the adaptation of CRISPR/Cas technology to develop new herbicide-resistant lines [98], which are mentioned in Table 4.

The three polyamine uptake transporter (PUT) genes in rice, including *OsPUT1*, *OsPUT2* and *OsPUT3*, homologous to the Arabidopsis *AtRMV1*, were successfully mutated using CRISPR/Cas9, resulting in increased resistance to the herbicide paraquat without any yield penalty [99]. A significant chromosomal fragment inversion (911 kb) and a 338 kb duplication were induced in the elite rice variety Jingeng 818 using CRISPR-Cas9. This process replaced the native promoters of *PPOI* and *HPPD* in rice, which are typically expressed at low levels in leaf tissue, with strong promoters of other linked genes in the mutant lines. As a result of these structural modifications, the *OxPPOI* and *OxHPPD* genes are highly expressed, conferring resistance to the herbicides FCD and bipyrazone [100].

## 4. Physiological Traits

Nitrogen, phosphorus and potassium are vital macronutrients for rice, with nitrogen fertilizer consumption at 15%, phosphorus at 13% and potassium at 11% of all fertilizers [101]. Photosynthesis in rice plants directly impacts yield by providing metabolic energy. Optimizing nutrient use efficiency (NUE) and enhancing photosynthetic efficiency are crucial for boosting productivity. Improved NUE maximizes nitrogen utilization, minimizing environmental losses, while enhanced photosynthesis drives growth and development by converting solar energy into chemical energy. Understanding the molecular mechanisms behind these processes is essential for sustainable increases in rice yield [12].

### 4.1. Genes Controlling NUE in Rice

Ammonium (NH_4_^+^) and nitrate (NO_3_^−^) are the two major nitrogen forms that can be available to plants [102]. *OsHHO3* belongs to the NIGT1/HHO subgroup within the GARP/G2-like transcription factor family found in rice, which comprises a nitrate-inducible NIGT1 protein and four nitrate non-inducible HHO proteins in rice [103]. The knockout of this gene via the CRISPR/Cas9 system generated seedlings with enhanced growth and increased shoot and root dry mass due to the increase in chlorophyll content and maximum quantum yield of photosystem II under N-deficient conditions. Transcriptome analysis of these mutant seedlings revealed an increase in the expression of genes encoding ammonium transporter (AMT) and nitrate transporter (NRT), as well as other genes related to nitrogen assimilation, in the roots of oshho3-KO mutants. Within these overexpressed genes, the upregulation of the three *AMT1* genes, namely *OsAMT1.1, OsAMT1.2* and *OsAMT1.3,* is very critical due to their influence on 95% of ammonium uptake in rice [104]. Thus, the knockout of *OsHHO3,* a transcriptional repressor of AMT1 genes, acts as negative regulator of nitrogen use efficiency in rice [105]. Table 5 enumerates the genes governing NUE, analyzed by CRISPR/Cas9.

### 4.2. Genes Controlling Photosynthetic Efficiency in Rice

Photosynthesis takes place in the chloroplast, whose function and development are governed by many genes. RNA editing plays a crucial role as a post-transcriptional process in plant organelles [108]. Among them, PPR (Pentatricopeptide repeat) is a major protein coded by the *OsPPR9* gene, participating in RNA editing [109]. Knockout of the gene through the CRISPR/Cas9 system showed decreased expression of genes involved in chloroplast development and proteins related to photosynthesis. Additionally, *OsPPR9* effectively interacts with *OsMORF2* and *OsMORF9*, genes that encode multiple organellar RNA editing factor (MORF) proteins which are significant for RNA editing [110,111]. Table 6 lists the genes that regulate photosynthetic efficiency which have been examined by CRISPR/Cas9.

## 5. Targeting Regulatory DNA Regions for Improving Rice Yield

**Figure 2:**
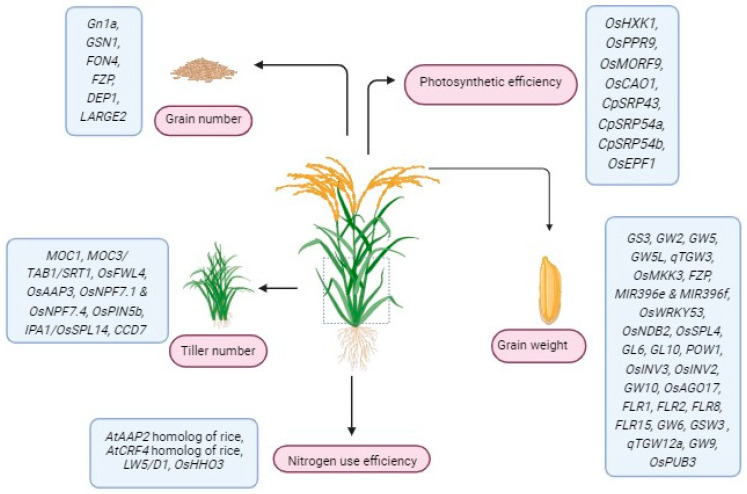
Applications of CRISPR/Cas9 in rice yield genes.

Genome editing tools have provided precise and efficient approaches for editing target genes, and substantial advancements have been achieved in editing the protein-coding part of the target gene. However, studies pertaining to the modifications of non-coding DNA regions with regulatory roles significantly trail behind. The non-coding parts of DNA, including those capable of being transcribed into miRNAs and long non-coding RNAs (lncRNAs), along with *cis*-regulatory elements (CREs) such as promoters, enhancers, silencers, transcription factor binding sites and introns, assume indispensable functions in the regulation of plant growth and development [117,118]. Promoters are crucial in regulating when, where, and how strongly genes are expressed. They contain regulatory elements that serve as binding sites for proteins such as transcription factors and RNA polymerases. Genome editing can modify promoter regions, allowing precise control over gene expression, which can impact rice traits like yield (Table 7), quality and biotic and abiotic stress resistance [119]. So, targeting these regions of a gene for editing will expand the application of genome editing tools in rice. Figure 2 highlights the yield-related genes edited by genome editing technologies.

## 6. CRISPR-Cas: Advantages over Other Gene Editing Tools

ZFNs and TALENs consist of a DNA-binding domain paired with the FokI enzyme, requiring extensive protein engineering to target new sequences. In contrast, the CRISPR/Cas9 system uses the Cas9 protein, which is guided by RNA to bind specific target sequences, allowing for simpler targeting by modifying the guide RNA (sgRNA) rather than engineering proteins [126]. Additionally, multiple sgRNAs can function with the same Cas9 protein, enabling simultaneous targeting of diverse DNA sequences, greatly enhancing the flexibility, efficiency, and versatility of genome editing with CRISPR/Cas9 [127]. CRISPR is compatible with various delivery methods, including plasmids, viral vectors, and ribonucleoprotein (RNP) complexes. CRISPR’s ability to be delivered as RNP complexes allows for a more immediate and transient expression of the editing compo-nents, which can reduce off-target effects and improve editing efficiency [128]. On the other hand, ZFNs and TALENs face limitations in delivery options and may have lower efficiency when delivered as proteins due to their larger size and complex assembly [129]. CRISPR/Cas9 demonstrates superior efficiency compared to ZFNs and TALENs, with reported editing activity ranging from 40 to 50% on average [130,131] and some studies showing rates as high as 73% in vitro [132]. Efficiency varies based on the specific Cas variant, sgRNA design, delivery method, and target site.

To broaden the versatility and to increase the range of applications for CRISPR technology, various advanced CRISPR-Cas tools, including base editing, prime editing, different Cas proteins, and multiple Cas9 orthologues, have been developed and successfully applied across a wide range of fields. Table 8 provides an overview of various Cas proteins, highlighting their distinct features and applications. For instance, Cas9 is widely used for its ability to introduce double-strand breaks in DNA, while Cas12a targets “AT”-rich regions in the genome, unlike Cas9, which typically targets GC-rich sequences. Cas13, on the other hand, specializes in RNA targeting, and Cas14 is known for its small size and potential for precise DNA editing. dCas9, a deactivated form of Cas9, is applied in gene regulation without cutting DNA, making it ideal for transcriptional modulation.

Base editing (BE) is an innovative and versatile genome editing (GE) system that allows for precise and highly predictable nucleotide modifications at genomic targets without the requirement for donor DNA templates, DSBs or dependence on HDR and NHEJ [138]. A base editor (BE) is formed by combining a catalytically inactive variant of Cas9 with a deaminase domain that targets either cytosine or adenine. Adenine base editors (ABEs) enable the conversion of an A-T base pair into a G-C pair, while cytosine base editors (CBEs) facilitate the transformation of a C-G base pair into a T-A pair [139]. Adenine base editing has been successfully applied to the yield gene OsSPL14 in rice, which is crucial for plant architecture and tiller number [140]. Prime editing is another GE technology that can effectively introduce all 12 possible types of point mutations, along with small insertions and deletions, in a precise and targeted way with advantageous editing to indel ratios. Prime editors are hybrid proteins that combine a Cas9 nickase domain (an inactive HNH nuclease) with an engineered reverse transcriptase domain [141,142].

**Figure 3:**
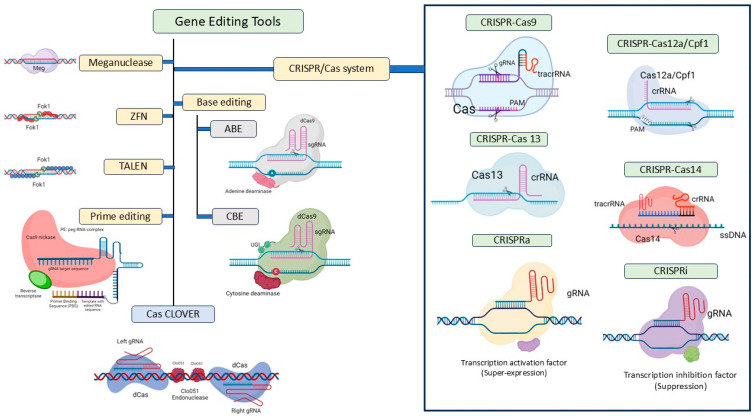
Different gene editing tools in rice. ZFNs—zinc finger nucleases, TALENs—transcription activator-like effector nucleases, CRISPR/Cas—clustered regularly interspaced palindromic repeat-associated Cas9 system, CRISPRa—CRISPR activation, CRISPRi—CRISPR interference, ABE—adenine base editing, CBE—cytosine base editing.

Another key advantage of CRISPR/Cas9 and CRISPR/Cpf1 is their simplicity in multiplexing, especially when compared to ZFNs and TALENs [143]. Multiplexing using CRISPR allows for the simultaneous targeting of multiple genes or genomic loci within a single experiment, significantly enhancing the efficiency of genome editing. This approach enables researchers to study complex genetic interactions and perform comprehensive modifications, facilitating advancements in functional genomics and crop improvement. CRISPR/Cas9-based multiplex editing of quantitative trait loci (QTLs), specifically *OsGS3, OsGW2* and *OsGn1a*, was performed across three elite rice varieties, J809, L237 and CNXJ, resulting in the successful generation of all seven combinations of single, double, and triple mutants for these targeted genes. Comprehensive analysis of these mutants revealed distinct effects on yield-related traits, including grain length, width, number, and 1000-grain weight. Notably, the triple mutants demonstrated a substantial increase in yield per panicle, with J809 achieving a remarkable 68% enhancement and L237 showing a 30% improvement [144]. Table 9 enumerates the advanced genome editing tools that have been utilized for the analysis of rice yield-related genes. Figure 3 presents the various gene editing tools including Cas variants, along with their advanced versions specifically available for use in plants, particularly rice. In summary, CRISPR-Cas technology provides significant advantages over other genome editing tools, including improved precision, simplified multiplexing and efficient mutation generation, making it a powerful platform for genetic research and crop enhancement.

CRISPR/Cas12a-RNP is an advanced genome editing system that utilizes the Cas12a protein complexed with ribonucleoprotein (RNP) to achieve precise modifications in plant genomes. This approach combines the targeting capabilities of CRISPR technology with the transient delivery of Cas12a and gRNA, allowing for efficient and specific editing while minimizing off-target effects. Unlike traditional DNA delivery methods, CRISPR/Cas12a-RNP is transgene-free and can enhance editing efficiency through controlled dosages and multiplexed targeting, making it a versatile tool for plant biotechnology [145,146]. To assess the efficiency of different genome editing systems, a comparative study was performed using three Cas9 nucleases (WT Cas9, HiFi Cas9 and Cas9 D10A nickase) along with two Cas12a nucleases (AsCas12a and LbCas12a) targeting the rice phytoene desaturase (*PDS*) gene. The results revealed that the delivery of WT Cas9, HiFi Cas9 and LbCas12a led to targeted mutagenesis, with LbCas12a demonstrating superior editing efficiency compared to both Cas9 variants. Editing with Cas9 primarily resulted in small insertions or deletions (indels) of 1–2 bp and larger deletions of 20–30 bp, frequently accompanied by the loss of the PAM site. Conversely, LbCas12a editing produced deletions ranging from 2 to 20 bp without affecting the PAM site. In summary, LbCas12a RNP complexes achieved a higher frequency of targeted mutagenesis at the *OsPDS* gene than AsCas12a or Cas9 RNPs [147].

**Table 9:** Different gene editing tools are employed to target rice yield genes.

Gene	Gene Editing Tool	Trait	Reference
*OsSPL14*	Base editing	Tiller number	[140]
*OsEPFL9*	CRISPR-LbCpf1 (*Lachnospiracae bacterium* Cpf1), CRISPR-FnCpf1 (*Francisella novicida* Cpf1)	Stomatal development	[148,149]
*OsDEP1*	CRISPR-LbCpf1, CRISPR-FnCpf1	Plant architecture and grain number	[149,150,151]
*OsGS3*	CRISPR-LbCpf1	Grain size	[152]
*OsDEP1*	Prime editing	Plant architecture and grain number	[153]
*OsSPL14*	Prime editing	Tiller number	[153]
*OsEPFL9*	CRISPR/Cas12a-RNP	Stomatal development	[145]
*NRT1.1B*	Base editing	Enhanced nitrogen use efficiency	[154]
*C287*	Base editing	Herbicide resistance	[155]
*GL2/OsGRF4*, *OsGRF3*	Base editing	Grain size and yield	[156]
*OsACC-T1*	CRISPR–Cpf1-based base editing	Herbicide resistance	[157]
*OsALS*	Prime editing	Herbicide resistance	[158,159]
*TFIIAg5*, *OsSWEET11a*, *OsEPSPS1* and *OsALS1*	Multiplexing—quadruple prime editing	Broad spectrum resistance to bacterial blight and herbicide	[160]
*OsSPL13*, *OsSPL14* and *OsGS2*	Multiplex prime editing	Major yield traits—grain size and weight, plant architecture, tiller number and NUE	[160]
*OsSWEET14*	Base editng (CBE)	Resistance to bacterial blight	[161]
*OsDEP1*, *OsNRT1.1b*, *OsWaxyT1*, *OsWaxyT2* and *OsWaxyT3*	Adenine base transition editor (ABE8e)	Panicle architecture, NUE, starch biosynthesis	[162]
*OsYSA*, *OsNAL*, *OsMIR396e*, and *OsPYL6*	CRIPSR/Cas12i3-based multiplex direct repeat (DR)-spacer Array Genome Editing system (iMAGE)	Chloroplast development, grain number, yield	[163]
*OsACCase*	Deactivated Cas12i3 base editor	Resistant to sethoxydim herbicide	[163]
*OsGRF4*	Prime editing	Grain yield	[164]

## 7. Off-Target Effects of CRISPR/Cas9 on Plant Physiology

The physiological impacts of CRISPR on plants largely stem from off-target mutations, which are a significant concern when evaluating the safety of genome-edited crops. These unintended genetic modifications can affect plant phenotypes or interact unpredictably with their environment. Reducing off-target frequencies is essential, and mismatches between the seed sequence (the 12 base pairs adjacent to the PAM) and the target sequence are critical in achieving this reduction [165]. In a study, multiplex CRISPR/Cas9 was employed to mutate genes like *OsPIN5b* (affecting panicle length), *GS3* (influencing grain size) and *OsMYB30* (related to cold tolerance). The editing efficiency across target sites ranged between 42% and 66%. Furthermore, eight triple mutants were developed, six of which exhibited off-target effects [37]. These off-targets can be minimized by refining gRNA design to increase specificity (numerous sgRNA design tools, such as CGAT, CRISPR-P, CHOPCHOP and CRISPR, help identify specific sgRNA sequences to improve targeting and reduce off-target impacts) [166,167]. Additionally, novel genome editing systems are used, including nickase-Cas9, base editing (C to T, A to G), CRISPR-Cpf1-RNP (recombinant CRISPR-Cpf1 ribonucleoprotein), tru-gRNAs (shorter/truncated guide RNAs for on-target site), SpCsa9-HF1 (high-fidelity engineered variants SpCas9), eSpCsa9 (enhanced specificity of SpCas9), Hypa-Cas9 (hyper-accurate Cas9 variant), C2c2/Cas13, SauCas9, C2c1, evoCas9 (evolved Cas9) [168]. For instance, the cytidine base editor (CBE) developed mutants resistant to African *Xanthomonas oryzae* pv. *oryzae* (Xoo) strains by targeting the *SWEET14* gene in rice with no off-targets [161]. Another study demonstrated that CRISPR-Cpf1 successfully induces targeted genome mutagenesis in rice for the *OsPDS* and *OsBEL* genes without any off-target effects. This indicates that, with careful selection of target sites, the CRISPR-Cpf1 system exhibits high specificity in vivo [169]. Similarly, a truncated gRNA (tru-gRNA)/Cas9 strategy successfully produced new alleles for the proton pump gene *OST2* in Arabidopsis without off-target effects. Monitoring the expression of Cas9 and tru-gRNA revealed a high mutation rate of 32.8% in transgenic plants, with no off-target impacts observed under a constitutive promoter [170]. These systems have been employed to reduce off-target editing, although research on them in plants lags far behind.

## 8. Challenges and Future Prospects in Genome Editing

Genome editing has demonstrated its effectiveness as a tool for enhancing crops throughout the preceding decade. However, along with its beneficial features, it also presents certain challenges. Mitigating these challenges can expand their applications in plant breeding programs. The first challenge resides in the development of efficient transformation systems for various crop species. The second challenge is to reduce the requirement of a unique canonical NGG PAM (protospacer adjacent motif) site by the SpCas9 (wild-type) system, which limits the flexibility and target site of the system [171]. Nevertheless, the identification of alternative PAM sites (NAG, NGA, etc.) and Cas variants like SaCas9 (*Staphylococcus aureus* Cas9) can promote the applications of genome editing [172,173]. The development of Cas9 variants such as VQR (NGA PAM) and VRER (NGCG PAM) has broadened the range of genome editing in rice [174,175]. Aside from these, it has been reported that the wild-type SpCas9 in itself is effective in targeting both NGG and NAG PAM sites in rice with high efficiency and relatively low off-targets in rice [176]. SpRY, another new variant of SpCas, was created to greatly increase BE (base editing) editing capabilities to almost PAMless [132]. In rice, scientists have made attempts to use SpRYn-CBE (cytosine base editor) and SpRYn-ABE (adenine base editor) and revealed the effective editing activities of SpRY on various PAM sites, including NCN, NTH, NAG, NAC, NAB, NCR, NTK and NGV, thus presenting them as promising alternatives in genome editing [177]. All these developments will contribute to widening the scope of genome editing in most cereal crops, especially rice, by using different engineered Cas9 variants with distinct PAM specifics. However, there is still a need for the development of more Cas9 variants to target a diverse array of PAM sites, as not all Cas9 variants work well with the plant system.

The third challenge is to minimize off-targets, as these unintended modifications can result in various non-quantifiable cellular signaling changes and physiological impacts in plants. To minimize off-target effects, several strategies are employed, including optimizing the design of guide RNAs to enhance specificity, using Cas proteins with higher accuracy (such as Cas9 variants like SpCas9-HF1 or eSpCas9), employing paired nickases and utilizing high-fidelity base editors [165]. Following off-targets, the next issue to be addressed is the system’s precision, which can be attained by targeting genes using HDR. The efficacy of the HDR pathway is comparatively low in plants, and their constraint lies in the lack of an effective delivery system for DNA template repair [139]. The next challenge is gaining widespread acceptance of gene-edited crops among consumers and regulators. Despite their potential benefits, public concerns about safety and ethics persist. Addressing this requires transparent communication, robust regulatory frameworks, and clear demonstrations of benefits to farmers and consumers, alongside efforts to build trust through education [178]. In CRISPRa, CRISPR activation gene transcription is boosted by fusing dCas9 (catalytically inactivated Cas variants (dCas9)) with activators or using scaffold RNA to recruit them. In CRISPRi, CRISPR interference dCas9 binds the TSS with inhibitors like KRAB or SRDX, disrupting transcription factor and RNA polymerase activity to regulate gene expression [179,180]. Cas-CLOVER is one of the advanced gene editing technologies that can be effectively utilized for precise genome modifications and targeted gene editing in various organisms, including plants. CLOVER is a dual-guided system that induces double-strand breaks through the dimerization of its nuclease components, and this technique has been successfully applied in bananas [181,182]. Therefore, it holds potential for exploitation in rice as well. Multiplex genome editing has been used to alter many genes at once and to understand the interaction among genes, as many yield traits in rice are interconnected [183]. However, the number of targets that genome editing tools can manipulate simultaneously is limited. Sequential editing [184] can be conducted to overcome these constraints.

## 9. Conclusions

Genetic editing in rice, with specific emphasis on genes linked to yield characteristics, possesses the potential to accelerate the process of rice breeding, considering the advancement in genome editing techniques and the emergence of novel discoveries, indicating a pivotal role in hastening crop improvement. By leveraging genome editing tools, especially the CRISPR/Cas9 system, researchers can precisely modify the candidate genes governing yield determination. The benefits of genome editing tools include excising transgenes from the genome by employing genetic segregation, leaving the resulting gene-edited plants entirely indistinguishable from those produced through traditional breeding methods. Understanding the complex genetic networks involving major yield traits and their responses to various environmental factors by integrating them with different omics approaches can provide comprehensive insights into the molecular mechanisms underlying the yield genes and enable researchers to create novel alleles in rice research. Investigating the role of non-coding regions, such as promoters, enhancers and non-translated regions, in regulating yield genes can aid in understanding gene expression and the respective yield modifications. Comprehensive studies analyzing the simultaneous modification (multiplexing) of several characteristic genes to optimize rice yield are still lacking. Future research should prioritize comprehensive studies aimed at understanding epistatic interactions among different yield-related genes. Overall, integrating genome editing tools such as the CRISPR/Cas9 system into rice breeding programs offers a transformative pathway towards sustainable enhancement in rice production.

## Figures and Tables

**Table 1:** CRISPR/Cas9 system for analyzing genes controlling rice grain number.

Gene	Coding Product/Protein	Modification	Pathway	Phenotype	Reference
*Gn1a*	Cytokinin oxidase/dehydrogenase	Gene disruption	Cytokinin biosynthesis	Increased panicle size and flower number per panicle	[17,18]
*GSN1*	OsMPK1 (Mitogen-activated protein kinase (MAPK) phosphatase enzyme)	Gene disruption	MAPK signaling pathway	Denser panicles and smaller grains	[21]
*FON4*	Receptor-like kinase	Gene Knockout	CLV pathway	Increased spikelet number per panicle	[23,29]
*FZP*	AP2/ERF transcription factor	Gene disruption	Different phytohormone-mediated signaling pathways	Increased grain numbers	[26]
*DEP1*	G-protein γ subunit	Gene disruption	G-protein signaling pathway	Dense erect panicle and increased grain number and density	[18,28]
*LARGE2*	HECT-domain E3 ubiquitin ligase OsUPL2	Gene disruption	Functions with APO1 and APO2 (positively regulates grain number and panicle number)	Large panicles with increased grain number	[30]

**Table 2:** CRISPR/Cas9 system for analyzing genes controlling rice grain weight.

Gene	Coding Product/Protein	Modification	Pathway	Phenotype	Reference
*GS3*	G protein γ subunit	Gene disruption	G-protein signaling	Increased grain size and quality	[18,37,38,43]
*GW2* (GRAIN WIDTH and WEIGHT2)	RING-type E3 ubiquitin ligase	Gene knockout	Ubiquitin-proteasome pathway	Improved grain filling and larger grain architecture	[44]
*GW5*	Calmodulin binding protein	Gene knockout	BR signaling	Increased grain width and weight	[40]
*GW5L*	Calmodulin binding protein	Gene knockout	BR signaling	Increased grain width and weight	[41]
*qTGW3*	OsSK41/OsGSK5	Gene disruption	Auxin signaling	Larger grain size	[42]
*OsMKK3*	Mitogen-Activated Protein Kinase Kinase 3	Gene knockout *	MAPK signaling	Decreases grain length	[45]
*FZP*	ERF domain	Gene knockout *	Ethylene biosynthesis	Smaller grains and degenerated sterile lemmas	[46]
*MIR396e* and *MIR396f*	Transcription factor	Gene disruption	GA biosynthesis	Increased grain size and altered plant architecture	[47]
*OsWRKY53*	Transcription factor	Gene disruption *	BA signaling and MAPK cascades	Smaller grains	[48]
*OsNDB2*	Type II NADPH dehydrogenase	Gene knockout	Alternative respiratory pathway in mitochondria	Increased grain size and 1000-grain weight	[49]
*OsSPL4*	SQUAMOSA PROMOTER BINDING PROTEIN-LIKEs	Gene disruption	Transcription factor	Increased grain number and size	[3,50,51]
*GL6*	Plant AT-rich sequence- and zinc-binding (PLATZ) transcription factor	Gene disruption	RNA polymerase III transcription machinery	Short grains with increase in number	[52]
*GL10*	MADS56	Gene knockout *	Gibberellic acid (GA) signaling pathway	Shorter grain length, lower grain weight and delayed flowering	[53]
*POW1* (PUT ON WEIGHT 1)	Homeodomain-like protein	Gene disruption	BR pathway	Increased grain size and leaf angle	[54]
*OsINV3* and *OsINV2*	Invertase	Gene knockout *	Sucrose metabolism	Smaller grain size	[55]
*GW10*	Cytochrome P450 subfamily 89A2 homology protein	Gene knockout	BR pathway	Increased grain number with smaller grains	[56]
*OsAGO17*	Argonaute (AGO) protein—component of the RNA-induced silencing complex (RISC)	Gene knockout *	sRNA pathway	Decreased grain size and weight	[57]
*FLR1, FLR2, FLR8* *FLR15*	FERONIA-like receptor protein kinases (FER)	Gene disruption Gene disruption *	FER pathway	Larger grain size Smaller grain size	[58]
*GW6* (GRAIN WIDTH 6)	GA-regulated GAST family protein	Gene knockout *	Gibberellin pathway	Reduced grain size and weight	[59]
*GSW3* (GRAIN SIZE AND WEIGHT 3)	GTPase-regulated protein	Gene knockout	Gibberellin pathway	Increased grain length, width and 1,000-grain weight	[60]
*qTGW12a*	Multidrug and toxic compound extrusion (MATE) transporter	Gene knockout *	Various regulatory pathways	Reduction in grain weight	[61]
*GW9*	Nucleus-localized protein containing both C2H2 zinc finger (C2H2-ZnF) and VRN2-EMF2-FIS2-SUZ12 (VEFS) domains	Gene knockout/disruption	GW2 ubiquitination pathway	Large grains with increased plant height	[62]
*OsPUB3*	U-box E3 ubiquitin ligase	Gene knockout *	Ubiquitin-proteasome pathway	Decreased grain weight and size	[63]

Note: * represents positive regulation.

**Table 3:** CRISPR/Cas9 system for analyzing genes controlling rice tiller number.

Gene	Coding Product/Protein	Modification	Pathway	Phenotype	References
*MOC1*	GRAS family transcription factor	Gene knockout *	Transcription factor	Reduced tillering	[66]
*MOC3*/ *TAB1*/*SRT1*	Homeobox domain -containing protein	Gene knockout *	Transcription factor	Reduced tillering	[66]
*OsFWL4*	Cysteine-rich protein	Gene disruption	Transcription factor	Increased tiller number and grain length	[68]
*OsAAP3*	Amino acid permeases	Gene knockout	Amino acid transport (regulate the concentrations of amino acids)	Increased tiller number	[69,70]
*OsNPF7.1 and OsNPF7.4*	Nitrate and di/tripeptide transporter	Gene knockout (differential expression in the presence of nitrogen)	Nitrogen transport	Plant architecture, NUE and tiller number	[71]
*OsPIN5b*	Endoplasmic reticulum localized protein	Gene knockout	Auxin balance and transport	Longer balance and transport tiller numbers	[37]
*IDEAL PLANT ARCHITECTURE1 (IPA1)*/ *OsSPL14*	Squamosa promoter binding protein	Gene disruption	Strigolactone signaling pathway	Tiller number varied according to the changes induced in the OsmiR156 target region	[18,72]
*CCD7 (CAROTENOID CLEAVAGE DIOXYGENASE 7)*	Carotenoid cleavage dioxygenase	Gene disruption, gene knockout	Strigolactone biosynthesis	Increased tillering and reduced height	[73,74]
*TB1 (TEOSINTE BRANCHED1)*	TEOSINTE BRANCHED1/CYCLOIDEA/PROLIFERATING CELL FACTORS (TCP) family transcription factor	Gene knockout	Strigolactone signaling pathway	Dwarf phenotype with increased tiller numbers	[75]
*EHD1*	B-type response regulator	Gene disruption	Flowering pathway	Enhanced yield and improved grain quality	[76]

Note: * represents positive regulation.

**Table 4:** CRISPR/Cas9 system for analyzing genes controlling stress and herbicide resistance in rice.

Trait	Gene	Gene Function/Coding Product	Improved Phenotype	Reference
**Biotic stress tolerance/resistance**	*OsSWEET13*	Sucrose transporter gene	Bacterial blight disease resistance	[84]
	*OsERF922*	Ethylene responsive factors	Increased resistance to blast disease	[85]
	*OsSWEET11*, *OsSWEET13*, *OsSWEET14*	Sucrose transporter genes	Broad-spectrum resistance to bacterial blight	[86]
	*ALB1*, *RSY1*	Melanin biosynthetic polyketide synthase	Rice blast resistance	[87]
	*Xa13*	Recessive resistant allele of *Os8N3*, a member of the *NODULIN3 (N3)* gene family	Bacterial blight disease resistance	[88]
	*eIF4G*	Translation initiation factor 4 gamma gene	Resistance to Rice Tungro Spherical Virus (RTSV)	[89]
	*OsCPR5.1*	Nucleoporin	Resistance to Rice Yellow Mottle Virus (RYMV)	[90]
**Abiotic stress tolerance/resistance**	*OsERA1*	β-subunit of farnesyltransferase	Drought tolerance	[91]
	*OsDST (Drought and salt tolerance)*	DST protein	Drought and salt tolerance	[92]
	*OsNAC006*	NAC transcription factor	Heat tolerance	[93]
	*OsPRP1*	Proline-rich protein	Cold tolerance	[94]
**Herbicide resistance**	*OsALS*	Acetolactate synthase	Significant tolerance to herbicides	[95]
	*OsEPSPS*	5-enolpyruvylshikimate-3-phosphate synthase	Resistance to glyphosate	[96]

**Table 5:** CRISPR/Cas9 system for analyzing genes controlling NUE.

Gene	Coding Product/Protein	Modification	Function	Phenotype	Reference
*AtAAP2* homolog of rice *AtCRF4* homolog of rice	Amino acid permease Transcription factor	Gene knockout	N transportation N uptake in roots	Shorter plants, increased panicle numbers, and dry biomass weight	[106]
*LW5/D1*	α subunit of G-protein	Gene disruption	N uptake and transport	Affect plant architecture and grain size by regulating N-transfer	[107]
*OsHHO3*	NIGT1 family protein	Gene knockout	Nitrate signaling	Enhanced growth and increased shoot and root dry mass	[105]

**Table 6:** CRISPR/Cas9 system for analyzing genes controlling photosynthetic efficiency.

Gene	Coding Product/ Protein	Modification	Pathway/Function	Phenotype	References
*OsHXK1*	Hexokinase	Gene knockout	Different phytohormone-mediated signaling pathways	High photosynthetic efficiency and yield	[112]
*OsPPR9*	DYW-PPR (Pentatricopeptide repeat)	Gene disruption *	RNA editing	Affects chloroplast growth and development	[111]
*OsMORF9*	MORFs (multiple organellar RNA editing factors)	Gene knockout *	RNA editing	Biogenesis of chloroplast ribosomes and chloroplast development	[113]
*OsCAO1*	Chlorophyllide a oxygenase	Gene knockout *	Chlorophyll degradation and ROS scavenging	Degradation and ROS senescence	[114]
*CpSRP43*, *CpSRP54a*, *CpSRP54b*	CpSRP (signal recognition particle)	Gene knockout	CpSRP pathway	Increased photosynthesis per photon absorbed	[115]
*OsEPF1*	Epidermal patterning factor	Gene knockout	Stomatal development and patterning	Enhanced the stomatal conductance and photosynthetic efficiency	[116]

Note: * represents positive regulation.

**Table 7:** Modifying regulatory gene regions to enhance rice yield.

Gene	Modification	Improved Phenotype	Reference
*OsCPK18/OsCPK4*	Modification of the phosphorylation motif of OsCPK18/OsCPK4	Improved disease resistance and yield	[120]
*OsPDCD5*	Improved plant architecture (plant height, panicle type, grain shape)	Enhanced yield	[121]
*OsSPL16*	Upregulation of pyruvate enzymes and cell cycle enzymes	Larger grain size and increased yield	[122]
*(Ehd1) Early heading date 1*	Modification of promoter at multiple sites	Delayed heading date and improved yield-related traits	[123]
*OsPYL9* *(Pyrabactin resistance 1-like 9)*	Upregulation of circadian rhythm and abiotic stress-responsive proteins	Increased yield and drought tolerance	[124]
*FZP*	Deletion of −157 to −45 bp in UTR (untranslated region) upstream of FZP 5′	Grain number	[26]
*IPA1/OsSPL14*	Deletion of An-1 (transcription factor) binding site of IPA1 (54-base pair cis-regulatory region)	Balances the trade-off between grains per panicle and tiller numbers, leading to increased grain yield	[125]

**Table 8:** Comparison of various Cas proteins.

Cas Protein	Host	Target	PAM Site	Cut Type	Unique Features	Reference
**Cas9**	*Streptococcus pyogenes*	Double-strand DNA (dsDNA)	NGG	DSB	Versatile, suitable for multiplexing	[133]
**Cas12a (Cpf1)**	*Prevotella* and *Francisella 1*	dsDNA	TTTN (AT-rich region)	Staggered cut	Staggers DSBs and promotes HDR mechanism	[134]
**Cas12j2 (CasΦ)**	Huge phages	dsDNA	NTTV	Staggered cut	Small and compact	[135]
**Cas13a**	*Leptotrichia shahii*	Single-strand RNA	None	RNA cleavage	RNA targeting	[136]
**Cas14**	Uncultivated Archaea	Single-strand DNA (ssDNA)	None	ssDNA cleavage	Small and specific for ssDNA	[137]
